# Antonie van Leeuwenhoek (1632–1723): Master of Fleas and Father of Microbiology

**DOI:** 10.3390/microorganisms11081994

**Published:** 2023-08-02

**Authors:** Ulrich Kutschera

**Affiliations:** 1 AK Evolutionsbiologie, 79104 Freiburg im Breisgau, Germany; kutscherau@gmail.com; 2 I-Cultiver, Inc., Manteca, CA 95336, USA

The Dutch scientist and entrepreneur Antonie van Leeuwenhoek (1632–1723) was the first to discover and describe microorganisms (protists, bacteria), living beings he characterized as “animalcules” (little animals). Using single-lensed microscopes created for his own, private research, he was able to see and draw microbes for the first time in the history of biomedical sciences. As a result, he became later known as the “father of microbiology”.

In this Editorial, I want to commemorate the 300th anniversary of van Leewenhoek’s death at the age of 90 years by briefly analyzing and summarizing his scientific legacy in different branches of microbiology, from medical aspects (pathogenic microbes in all kinds of organisms) to symbiotic relationships. In addition, van Leeuwenhoek’s neglected agenda of “public understanding of microscopic biology” is outlined, with reference to his status as a key figure in one of E.T.A. Hoffmann’s famous romantic fairy tales of 1822, *Master Flea* (Figure 1).

On 20 April 2011, the Netherlands Society of Microbiology was established in Delft, Holland. As their first Chairman, the botanist Martinus W. Beijerinck (1851–1931) was elected, who later became famous as one of the discoverers of Tobacco Mosaic Virus-Particles, and hence as co-founder of the scientific discipline of *Virology*.

Twenty-two years later (1934), the re-named *Dutch Society for Microbiology* started to publish their own periodical, entitled *Antonie van Leeuwenhoek*. This “Journal of Microbiology” (sub-title) accepted articles on the “fundamental and applied aspects of microbiology, with a particular emphasis on the natural world”. In August 2023, Issue 8 of Vol. 116 of this well-established journal was published, now distributed via the Springer-Nature-Company.

Who was Anthonie van Leeuwenhoek—the man whose name lives on in the literature of the romantic era, and as the title of one of the well-known scientific journals dealing with microbes and their host organisms? Leeuwenhoek died 300 years ago, on 26 August 1723, at the age of 90 years and 10 months, in Delft, Dutch Republic. His life and achievements have been summarized in excellent monographs and articles, on which the following brief account is based [1,2].

Born on 24. October 1632 in Delft as the son of a basket manufacturer, Antonie later moved to Amsterdam to become the apprentice of a Scottish merchant, 1648 to 1653.

In 1654, the then 22-year-old man opened a draper’s shop in Delft. At that time, he married his first wife, Barbara de Mey, the daughter of a textile merchant. From the five children born to the couple, three sons and one daughter died as infants. His only surviving child, daughter Maria, later took care of her ageing father. Five years after the death of Barbara (1666), Antonie married his second wife, Cornelia. No surviving children are recorded from this relationship, that lasted until 1694. In 1669, Antonie van Leeuwenhoek passed the examination to become a land surveyor, and ten years later, he was appointed as a “Weights and Measures Inspector for the city of Delft”.

In 1670, the then 38-year-old craftsman manufactured his first single-lens microscopes and started his career as a research scientist, without ever gaining a “higher education”, or ever attending a university. In the following decades, he produced hundreds of lenses and ca. 25 different microscopes. Three years later (1673), Antonie van Leeuwenhoek began his correspondence with the Royal Society in London, which lasted over the next 50 years—until his death. In more than 300 letters, written in Dutch, van Leeuwenhoek summarized his experiments and microscopic observations in detail. These documents were translated into English and published by the society.

In 1674, Antonie van Leeuwenhoek observed for the first time red blood cells and protozoa; in 1676, the 44-year-old amateur naturalist discovered bacteria, and spermatozoa from the testes of an animal. In these pioneering studies, he used his custom-made microscopes, equipped with his own lenses (magnification up to 500-fold). At the age of 70, he retired from his municipal duties in Delft, but received a salary until his death. 

Much has been written about van Leeuwenhoek’s discovery of bacteria, as documented in his letter of 17 September 1683. In this report to the Royal Society, he described his microscopical observations on the plaque isolated from his own teeth: moving living “little animalcules” (bacteria), and other microorganisms. However, it remained largely unknown that Antonie van Leeuwenhoek was also an active “popularizer” of his scientific observations, leading to his fame as a key figure in E.T.A. Hoffmann’s fairy tale.

We know that the Dutch naturalist van Leeuwenhoek was heavily influenced by the British amateur scientist and polymath Robert Hooke (1635–1702), notably by Hooke’s popular book *Micrographia*, published in 1665 [1]. In this first “scientific bestseller”, Hooke, in 1665, not only defined the term “cell” (small chamber surrounded by walls), but also depicted numerous “little animals” well-known to the health and physical well-being of the general public, such as fleas, etc., in high-quality woodcuts. 

Thirty years later, Antonie van Leeuvenhoek published a similar book for the general reader, entitled *Arcana Naturae Detecta—1695* (in Latin) [3], which was designed to educate the public about his microscopic observations he submitted at regular intervals in the form of letters to the Royal Society in London (UK). In this comprehensive volume (600 pages), with the popular title “*Nature’s Mysteries Disclosed*”, van Leeuwenhoek described and depicted the development of a “flea”, from the egg via larval stages to an adult specimen, in remarkable detail. These splendid figures led to the 18th century saying that the microscopists, like van Leeuwenhoek, were “flea researchers”, or, in other words, microbiology was once associated with the elucidation of the fine structure of flea-like parasites of humans.

When the German universal genius Ernst Theodor Amadeus (E.T.A.) Hoffmann (1776–1822), who was a composer, author, painter and jurist in one person [4], designed his fairytale fantasy novel *Meister Floh* (Master Flea), he clearly had Antonie van Leeuwenhoek in mind, who was, in addition to Spallanzani, the key figure in this book (Figure 1). Since Hoffmann ironically described one of Prussia’s major police officers in the oppressive political system of his time, dedicated to maintaining the old authoritarian regime, he got into legal trouble. As a defender of free speech, the jurist Hoffmann had to accept the censorship of parts of the van Leeuwenhoek *Master Flea* novel; however, only one century after his death, the complete story was re-constructed and published in full [4].

Finally, I want to comment on Antonie van Leeuwenhoek’s scientific legacy. As the discoverer of bacteria, infusoria, micro-algae, spermatozoa, red blood cells, etc., he became known as the doyen of microbiology, once labelled as “flea-research”. Although van Leeuwenhoek did not accept a connection between microbial contamination of human tissues and diseases, his own medical problems during the final years of his life document that he was fully aware of human suffering and death. Obviously, his study and documentation of the ontogeny of common fleas (small flightless, blood-sucking, parasitic insects, order Siphonaptera) attests to his commitment to biomedical research. Based on these studies, he opposed the idea of “spontaneous generation”, and favored the concept that organisms originate from their parents.

Since Antonie van Leeuwenhoek studied plants, animals and human samples (plaque on his teeth) alike, he must be credited with the title “universal biologist”, equipped with an endogenous drive for insights. In his letter of 12 June 1716, van Leeuwenhoek pointed out that “my work, which I’ve done for a long time, was not pursued in order to gain the praise I now enjoy, but chiefly from a craving after knowledge, which I notice resides in me more than most other men”. As a result, the interdisciplinary branch of the biomedical sciences we today call “Microbiology” can be traced back to the experimental work of this Dutch businessman and naturalist, whose legacy not only lives on in the title of the journal *Antonie van Leeuwenhoek*, but also in the German romantic literature (Figure 1).

## Figures and Tables

**Figure 1:**
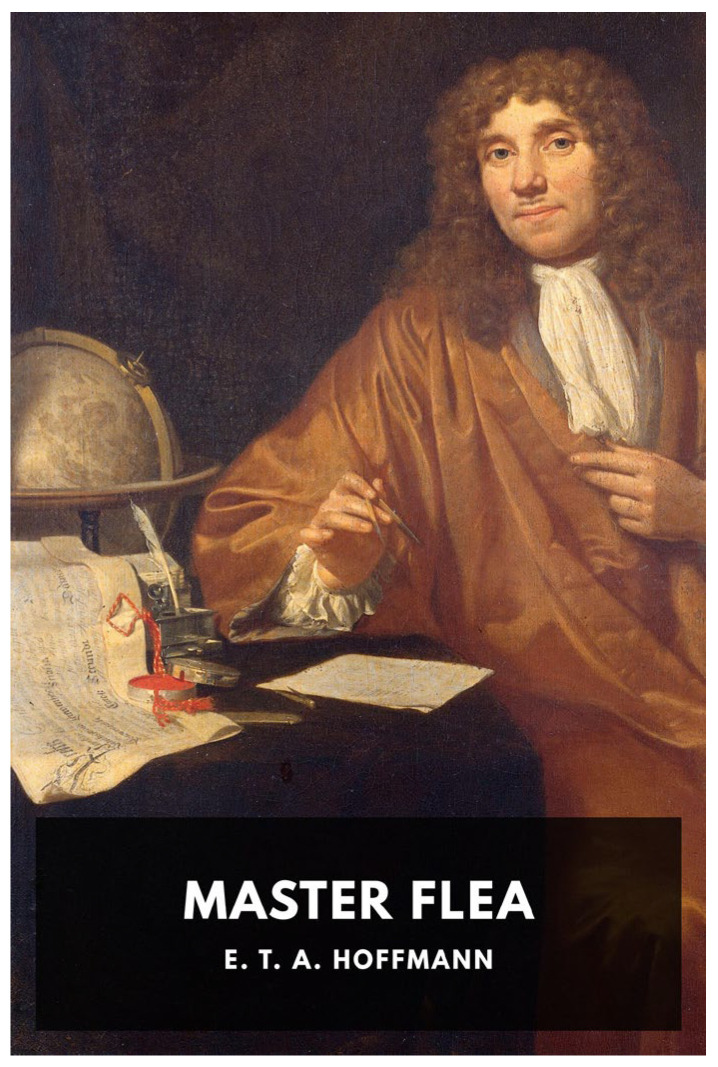
Portrait of Antonie van Leeuwenhoek (1632–1723), with scientific instruments (forceps, etc.) in his private laboratory, as depicted on the cover page of the English version of E.T.A. Hoffmann’s fantasy novel *Meister Floh* (*Master Flea*-1822-translated by George Soane). (Adapted from Standard EBooks, 2022, https://standardebooks.org/ebooks/e-t-a-hoffmann/master-flea/george-soane (accessed on 14. July 2023)).

## Data Availability

Not applicable.
